# A Tumor-Infiltration CD8+ T Cell-Based Gene Signature for Facilitating the Prognosis and Estimation of Immunization Responses in HPV+ Head and Neck Squamous Cell Cancer

**DOI:** 10.3389/fonc.2021.749398

**Published:** 2021-09-28

**Authors:** Yingning Wu, Lingzhang Meng, Kai Cai, Jingjie Zhao, Siyuan He, Jiajia Shen, Qiuju Wei, Zechen Wang, Suren Sooranna, Hengguo Li, Jian Song

**Affiliations:** ^1^ Medical Imaging Center, The First Affiliated Hospital of Jinan University, Guangzhou, China; ^2^ Center for Systemic Inflammation Research (CSIR), School of Preclinical Medicine, Youjiang Medical University for Nationalities, Baise, China; ^3^ Department of Radiation, The Affiliated Hospital of Youjiang Medical University for Nationalities, Baise, China; ^4^ Radiation Therapy Center, The First Affiliated Hospital of Guangxi University of Chinese Medicine, Nanning, China; ^5^ Life Science and Clinical Research Center, The Affiliated Hospital of Youjiang Medical University for Nationalities, Baise, China; ^6^ School of Pharmacy, Youjiang Medical University for Nationalities, Baise, China; ^7^ Department of Metabolism, Digestion and Reproduction, Imperial College London, Chelsea & Westminster Hospital, London, United Kingdom; ^8^ Department of Radiation Oncology, Renji Hospital, School of Medicine, Shanghai Jiao Tong University, Shanghai, China

**Keywords:** CD8+ T cells, HPV, immunization-correlated genes, immunization-correlated therapy, differentially expressed genes, predicted prognosis, head and neck squamous cell carcinoma

## Abstract

**Background:**

CD8+ T cells, which play a vital role in response to adaptive immunity, are closely related to the immunization responses to kill tumor cells. Understanding the effects exerted by tumor-infiltrated CD8+ T cells in HPV+ and HPV- head and neck squamous cell carcinoma (HNSCC) patients is critical for predicting their prognosis as well as their responses towards immunization-related therapy.

**Materials and Methods:**

HNSCC single cell transcriptome was used to screen for differentially expressed genes (DEGs) based on CD8+ T cells. A gene signature associated with CD8+ T cells was built and verified with the cancer genome atlas dataset with a view to predicting the prognosis of HNSCC patients. Risk scores were calculated for HNSCC cases and categorized into either high- or low-risk cohorts. The prognosis-correlated data of the risk scores were analyzed by using Kaplan-Meier survival curves and multi-variate Cox regression plots. In addition, the possibility of using the genetic profiles to predict responses toward immunization-related therapy was explored.

**Results:**

From the DEGs screened from the sequencing of single-cell RNA, a gene signature of 4 genes (ACAP1, ANKRD28, C12orf75, and M6PR) were identified. It was seen that these genes could predict overall survival in HPV+ HNSCC patients. In addition, high- and low-risk HPV+ HNSCC patients showed marked differences in their CD8+ T-cell infiltration due to immunization when clinical characteristics were taken into consideration. This correlated with their immunization therapy responses.

**Conclusions:**

Our work provides insights into explaining the restricted responses of current immunization checkpoint inhibiting substances in HPV+ HNSCC patients. A novel genetic signature to predict the prognosis and immunization-correlated therapeutic responses is presented. This will provide potential new therapeutic opportunities for HPV+ HNSCC patients.

## Introduction

Head and neck squamous cell carcinoma (HNSCC) refers to cancer of the oral cavity, oropharynx and larynx, and is the sixth commonest carcinoma globally (1). More than 550,000 new patients are reported each year, accounting for approximately 4% of carcinomas worldwide (2). Because most cases present with locally advanced disease, HNSCC is correlated with poor prognosis and results in high mortality (3). Conventional treatments exhibit limited effectiveness, and new therapeutic strategies capable of broadening the existing treatment options for HNSCC are urgently required (4). Recent clinical trials have demonstrated that programmed death ligand 1 (PD-L1) or programmed death 1 (PD-1) blockade exhibit clinically meaningful anti-tumor activity together with an acceptable safety profile when used in the treatment of HNSCC patients (5, 6). However, despite this progress, only about 20-30% of HNSCC cases survived after anti-PD-1/PD-L1 therapies, and the response towards PD-1/PD-L1 blockade is still far from satisfactory. Therefore, there is an urgent need to further understand the immunization state of the cell during this disease and identify features correlated to the response ability of existing immunization therapies, thereby paving the way to the development of new single and multi-drug immunization therapies.

HNSCC, is a type of carcinoma that can arise due to genetic alteration caused by either exposure to carcinogens (such as alcohol and/or tobacco) or *via* malignant conversion due to HPV infection (7, 8). There is an alarming growth of HPV + HNSCC in western countries, with up to half of all HNSCC patients residing in the US, as the presence of HPV infections is considered to increase the risk of the disease (9). However, HPV-correlated HNSCC is suggested to exhibit distinctive biological and clinically-associated characteristics, with the presence of HPV conferring a survival advantage when compared to its absence (10). Distinct tumor-infiltration immunization populations were identified in HNSCC patients, with a greater proportion of dysfunctional CD8+ T cells seen in HPV- HNSCC (11). A higher rate of response towards PD-1/PD-L1 was also identified in HPV+ patients compared to those who were HPV-. Thus, the presence of HPV may well be a factor that can be used to classify HNSCC. However, the underlying mechanisms and potential associations between the HPV state and the tumor immunization environment still needs to be characterized.

In the present study, we attempted to elucidate the correlation between the HPV state and immunization environment-related factors by using a multicenter database. A single-cell RNA sequencing dataset was used to assess the various subpopulations of immune cells and particular genes that may differ in HPV+ and HPV- HNSCC patients. Using a combination of RNA-seq data from a large number of HNSCC cases and their corresponding clinical information, a gene signature for tumor-infiltrated CD8+ T cells was established using multiple machine learning algorithms. This risk-associated gene signature was verified using the gene expression profiles and clinically associated information from an independent cancer genome atlas (TCGA) provisional dataset. The genetic signature obtained may provide future targets for increasing our knowledge of the mechanisms that govern HPV+ and HPV- HNSCC. This study may also increase immunization checkpoint blockade therapy efficacy with respect to treatment of this disease.

## Materials and Methods

### CD8+ T Cell Estimation in HNSCC Patients

The TIMER2.0 database (http://timer.comp-genomics.org/) was utilized to explore the relationship between tumor infiltration of CD8+ T cells and the prognosis of HNSCC in patients (12). We analyzed the immunization infiltration CD8+ T cells in different carcinoma types by multiple immunization deconvolution approaches, by using Cox regression correlation and Kaplan-Meier survival curves. These data obtained were used to correlate the prognosis data of the relevant immunization infiltration data in a range of carcinoma categories.

### Research of CD8+ T Cell-Correlated Immunization-Correlated Genes in HNSCC

Single cell transcriptomes include 130,721 cells from HPV- HNSCC and HPV+ HNSCC (10). Both cohorts consist of PBMC and tumor infiltrated leukocytes (TIL). In the current analysis we have only included 60,676 cells from the TIL.

### Single-Cell RNA-Seq Data Analysis

Specific to the integrated investigation of single-cell data, these were normalized with the SCTransform approach and then analyzed by conducting a mutual principal unit investigation (PCA) (https://satijalab.org/seurat/v3.1/integration.html) (13). The PCA analysis was also conducted on the integrated datasets with the cluster analysis being performed with uniform manifold approximation and projection (UMAP). Cluster analysis of single-cells was performed using Seurat’s graph-based clustering approach [R software package Seurat (version 2.3.4)] with the FindClusters feature resolution set to 0.1. Subsequently, the clusters were visualized by using the UMAP (version 0.2.6.0) graph. For quality control, unique molecular identifier counts of less than 500 and double multiples were removed. Furthermore, cells with > 5% mitochondrial genes and > 50% ribosomal genes were filtered out.

### Collection and Processing of the HNSCC RNAseq Dataset

The RNA sequencing dataset for HNSCC and the corresponding clinically related data originated from the TCGA database (https://portal.gdc.carcinoma.gov/), which consists of 279 samples. The validation cohort dataset is TCGA provisional database, consisting of 249 samples. The raw gene expression dataset was processed. Probe IDs received the annotation toward the gene from the corresponding platform annotation profile of the GDC website and the raw matrix data received the quantile normalization and log2 conversion. Samples with missing data were excluded.

### Building a CD8+ T Cell-Correlated Gene Signature

Single-cell data was classified into specific cell types and divided according to their respective tissue sources. The corresponding transcriptome investigation data were compared in order to screen for DEGs. To increase the efficiency of the study the candidate DEGs were taken as min.pct > 0.25 and | Log2 (FC) | > 0.5.

The association between HNSCC tumor-infiltration CD8+ T cell-correlated DEGs and overall survival time in TCGA HNSCC cases was studied. Univariate Cox regression analysis was carried out for identifying the genes associated with survival (p value < 0.05). Subsequently, the significance of candidate genes was selected using variable importance in a randomized survival forest (RSF) algorithm. A risk score model with the selected DEGs was built using multi-variate Cox regression approaches. In addition, the Kaplan-Meier test was employed for a number of gene features and p-values (log) were determined. Receiver operating characteristic (ROC) analysis was carried out for 3- and 5-year overall survival rates and area under the curves (AUCs) were determined for assessing the specificity and sensitivity of the gene signature. In addition, for testing the robustness of the results, the HNSCC tumor-infiltration CD8+ T cell-correlated gene signature was further verified with the TCGA HNSCC dataset.

### The Effects of Age, Gender, Alcohol and Smoking on HNSCC Patients

To assess the correlation of risk score distribution and clinically related characteristics, HNSCC patients were grouped according to age and gender as well as their status regarding alcohol consumption and smoking. In addition, risk scores were calculated in order to assess the patients’ prognosis for HNSCC in the presence and absence of HPV by using multi-variate Cox regression correlations.

### Statistical Analysis

Statistics investigations were carried out with R software (version 3.6.0). Kaplan-Meier tests and ROC analysis were performed with the “survivor” and “survROC” software packages (14). Optimal cutoff data points were calculated using the “survminer” package (15). Single-variate and multi-variate Cox regression correlations were used to assess the prognosis-correlated factors of interest. Hazard ratios (HR) and 95% confidence intervals (95% CI) were presented for all the prognosis-correlated factors. In statistical tests, P<0.05 was considered statistically significant.

## Results

### A Comparison of CD8+ T Cells in HPV− and HPV+ HNSCC Patients

**Figure 1:**
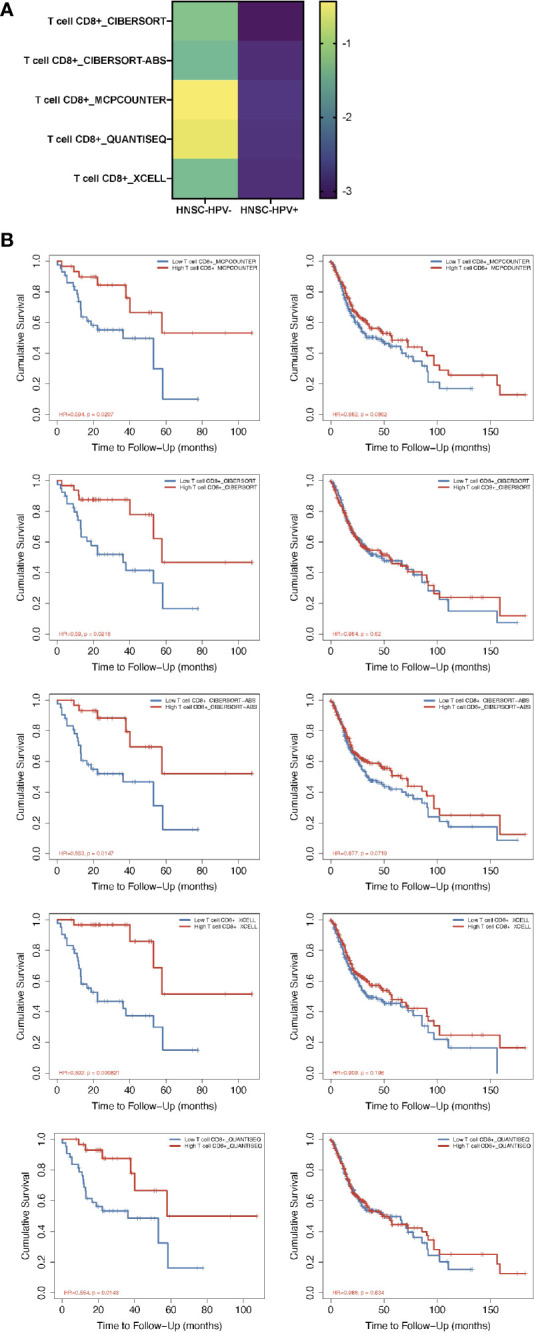
Prognosis-correlated data of CD8+ T cells from patients with HPV− and HPV+ HNSCC. **(A)** A heat map of data from the multiple-variate Cox proportional risk model in terms of CD8+ T cells from patients with HPV− *versus* HPV+ HNSCC. The z-scores represent the risk scores. **(B)** Kaplan-Meier survival analysis showing the levels of CD8+ T cells from patients with HPV− (right column) and HPV+ (left column) HNSCC by using MCPcounter, CIBERSORT, CIBERSORT-ABS, XCELL and QUANTISEQ approaches.

From the TIMER2.0 website, a number of immunization deconvolution approaches including “XCELL (16)”, “MCPCOUNTER (17)”, “QUANTISEQ (18)”, “CIBERSORT-ABS” and “CIBERSORT (19)” were employed for estimating immunization infiltration of CD8+ T cells in HPV− and HPV+ HNSCC patients. With the single-variate Cox proportional risk model, the found that the tumor-infiltrated CD8+ T cells were protective for cases with HPV+ HNSCC, but this was not seen in HPV− HNSCC patients (**Figure 1A**). According to the Kaplan-Meier curves obtained, the survival period of the high tumor-infiltration CD8+ T-cell cohort was significantly longer than that of the low CD8+ T tumor infiltration seen in HNSCC patients who also had HPV, irrespective of the deconvolution approach used (**Figure 1B**).

### Single-Cell RNA-Seq-Based DEGs Identification

**Figure 2:**
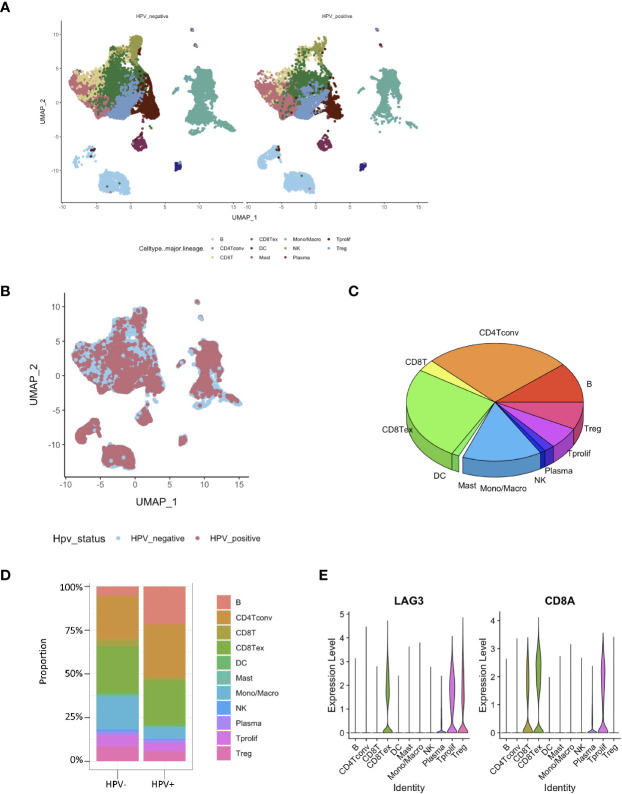
Identification of HPV− and HPV+ HNSCC tumor-infiltration of CD8+ T cell-correlated genes. **(A)** View of single cell samples from HPV− and HPV+ TIL. The annotated UMAP plot identifying 11 distinctive cell types. **(B)** The annotated UMAP plot of HPV− and HPV+ HNSCC TIL. **(C)** A pie chart of the seven cell types that make up the TIL of HNSCC. **(D)** Bar graphs of the cell proportions in the TIL of HPV− and HPV+ HNSCC. **(E)** Violin plots illustrating the expression of CD8A and exhausting T cell marker LAG3 in different TIL cell types.

The RNA-seq based on single cells consisted of 130,721 immunization cells from the tumor infiltrated leukocyte (TIL) samples obtained from HNSCC patients. Using the UMAP algorithm, this mixture of 11 cell types including CD8 T, CD4 T, dendritic, CD8+ T, mast, natural killer (NK) and plasma cells as well as mon/macrophages were unambiguously clustered and annotated (**Figure 2A**). Single-cell clustering was also based on the presence and absence of HPV (**Figure 2B**). According to the pie chart, the number of CD8+ T cells was an important unit of the HNSCC TILs (**Figure 2C**). The bar graphs also indicated that CD8+ T cells accounted for the greatest proportion of infiltration of all the immunization cells into the HPV− and HPV+ tumors in HNSCC patients (**Figure 2D**). The gene expression of LAG3 showed that exhausting T cells account for majority of the CD8 T cells (**Figure 2E**).

### Building a CD8+ T Cell-Correlated Gene Signature

**Figure 3:**
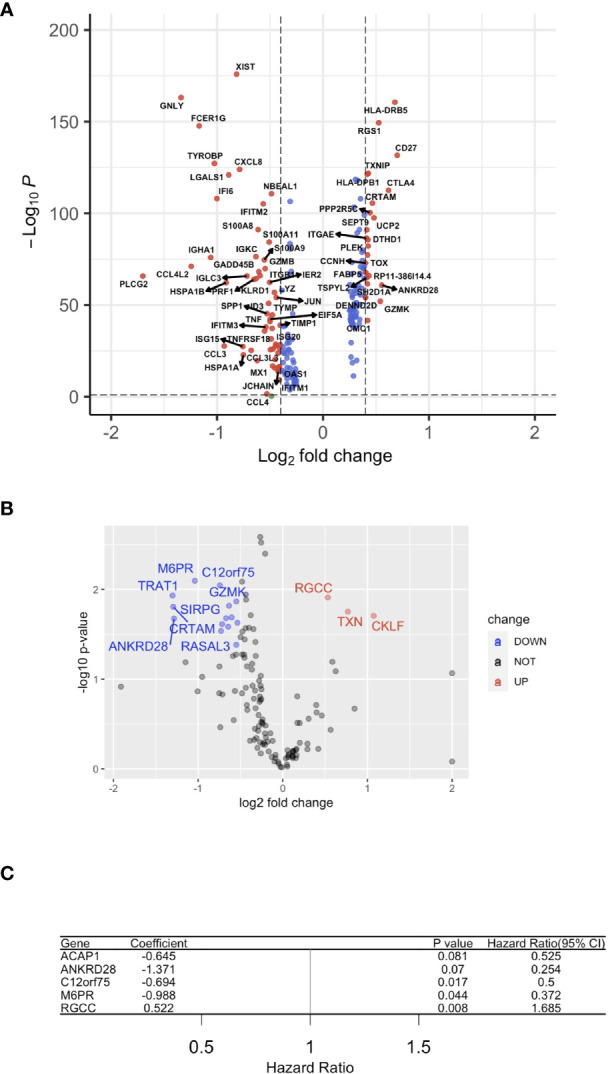
A gene signature using infiltrated CD8+ T cells. **(A)** A volcano plot of the differentially expressed genes (DEGs) between HPV+ and HPV− HNSCC tumor-infiltrated CD8+ T cells. **(B)** A volcano plot showing the DEGs obtained from Cox regression analysis of survival-related HPV+ HNSCC-infiltrated CD8+ T cells. **(C)** Forest plot lines of the top 4 genes screened by using random survival forest analysis of HNSCC patients.

Subsequently, the HPV+ HNSCC tumor-infiltration CD8+ T cell-correlated DEGs were screened based on the selection criteria in the approaches used (**Figure 3A**). To screen for the crucial survival-related factors, the DEGs from the CD8+ T cells of HPV+ HNSCC were analyzed using single-variate Cox regression for the TCGA dataset, and a total of 21 DEGs were identified to be significantly correlated to survival in these patients (p < 0.05) (**Figure 3B**). Based on the random forest algorithm, the top 5 significant genes, ACAP1, ANKRD28, C12orf75, M6PR and RGCC, were screened (**Figure 3C**).

**Figure 4:**
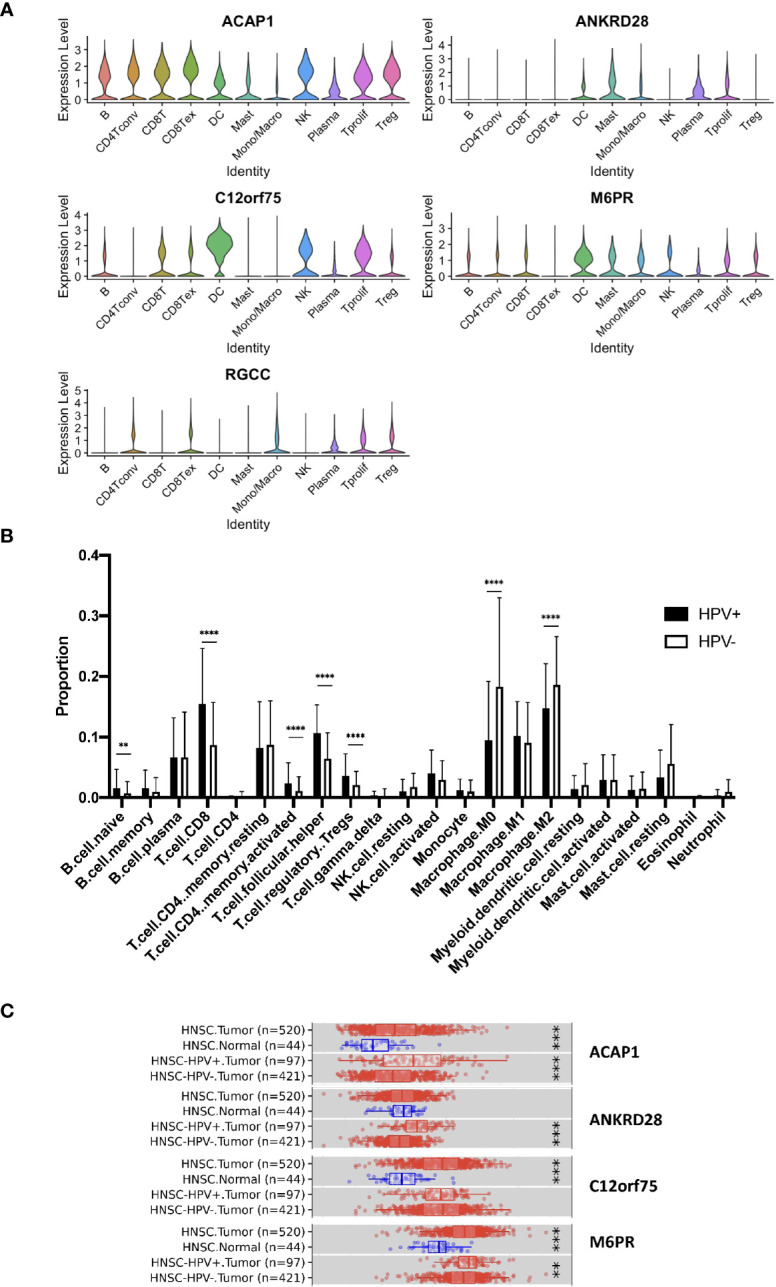
Expression of the signature genes. **(A)** Violin plots illustrating the expression of the survival related signature genes in different TIL cell types of scRNA data. **(B)** Bulk seq data were deconvoluted using CIBERSORT method. Bar graph illustrating the proportion of infiltrated immune cells of HPV+ and HPV− HNSCC. **(C)** Expression of signature genes were analyzed using TIMER2 website. Box charts showed the expression of the signature genes of the TCGA data. “**” refers p < 0.01, “***” refers p < 0.001 and “****” refers p < 0.0001.

Further, we studied the expression of the significant genes in different immune cell subsets and found the association of ACAP1, ANKRD28, C12orf75 and M6PR, but not RGCC, with CD8+ T cells (**Figure 4A**). Also, we checked the TCGA data using CIBERSORT deconvolution and TIMER2 website, which demonstrated a substantial fraction of CD8+ T cells and the 4 signature genes in HPV+ compared to HPV- HNSCC (**Figures 4B, C**). RGCC was detectable, and therefore excluded from the signature genes.

**Figure 5:**
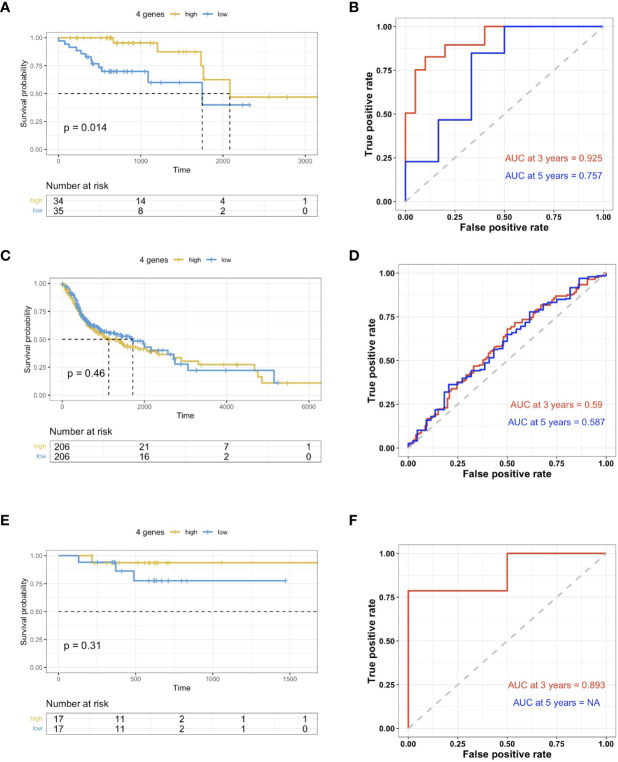
Validation of prognosis gene labels for HNSCC cases. **(A)** Kaplan-Meier (KM) analysis of the risk group that were defined with CD8+ T cell-correlated gene tags in the TCGA dataset for HPV+ HNSCC. **(B)** KM analysis of the risk group that were defined with CD8+ T cell-correlated gene tags in the TCGA dataset for HPV- HNSCC. **(C)** KM analysis of the risk group that were defined with CD8+ T cell-correlated gene tags in the TCGA provisional dataset for HPV+ HNSCC. **(D)** Three- and five-year ROC survival curves from the TCGA dataset for HPV+ HNSCC. **(E)** Three- and five-year ROC survival curves from the HPV- HNSCC TCGA dataset. **(F)** Three- and five-year ROC survival curves from the TCGA provisional dataset for HPV+ HNSCC.

The risk scoring system was then built using these 4 genes with multi-variate Cox analysis using the TCGA dataset. In accordance with the formula, a risk score was calculated for the respective cases. The HPV+HNSCC cases in the TCGA dataset were then divided into high-risk and low-risk cohorts with the optimal cutoff data for the risk scores. Kaplan-Meier curves showed that the high-risk group survived for longer periods in comparison with those patients in the low-risk cohort (**Figure 5A**). In comparison, there was no clear distinction in the high-risk cohort of HPV- HNSCC case survival (**Figure 5B**). This was further validated in the TCGA provisional dataset for HPV+ HNSCC (**Figure 5C**). To estimate the predictive power of genetic characteristics, ROC curve analysis of the HPV+ HNSCC cases were plotted and this showed an AUC of 1 and 0.739 for 3 year-survival (**Figure 5D**), while ROC survival curves for the HPV- HNSCC patients were less significant (**Figure 5E**). This was verified by using the TCGA provisional dataset which showed an AUC curve of 0.839 for 3 year-survival (**Figure 5F**).

### Correlation of Risk Score Distribution and Clinical Characteristics of HNSCC Patients

**Figure 6:**
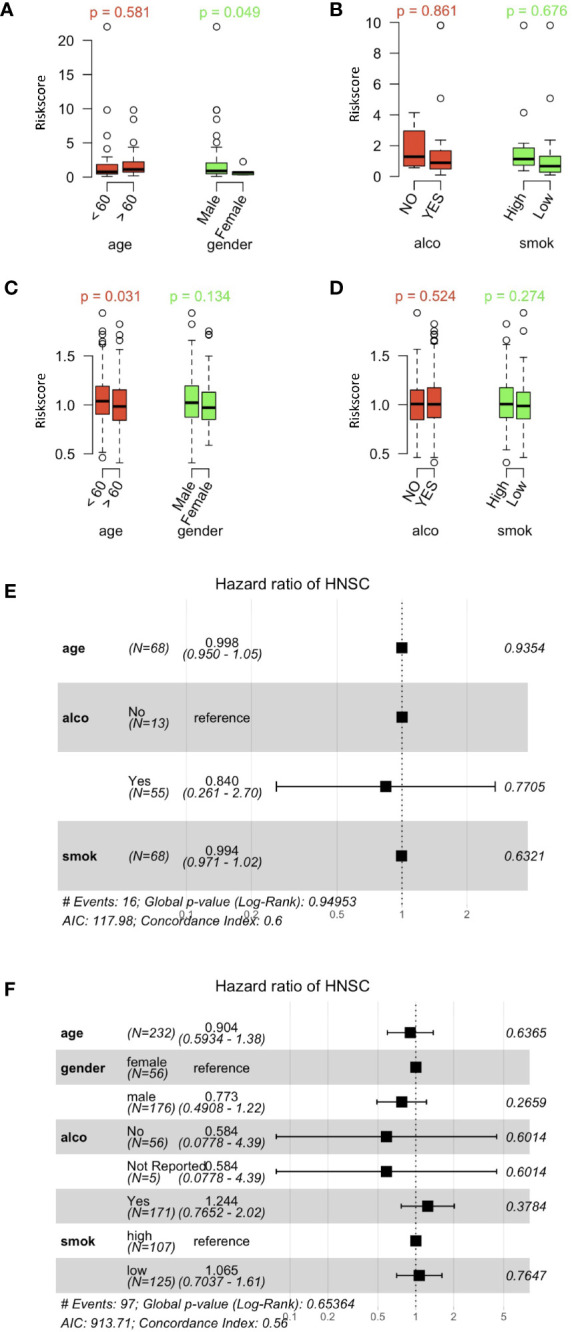
Correlation between risk scores and clinically related characteristics of HNSCC patients. **(A)** Distribution of risk scores obtained when patients’ data obtained from the HPV+ HNSCC TCGA dataset were separated by age and sex. **(B)** Risk score distributions for alcohol and smoking in the HPV+ HNSCC TCGA dataset. **(C)** Distribution of risk scores obtained when patients’ data obtained from the HPV- HNSCC TCGA dataset were separated by age and sex. **(D)** Risk score distributions for alcohol and smoking in the HPV- HNSCC TCGA dataset. **(E)** Multi-variate Cox regression forest plots of the risk scores and clinically related characteristics in the HPV+ HNSCC TCGA dataset. **(F)** Multi-variate Cox regression forest plots of risk scores and clinically related characteristics in the HPV- HNSCC TCGA dataset. Alco and Smok refer to alcohol consumer and smoker.

HPV+ HNSCC cases in the TCGA dataset were categorized according to high or low risk score cohorts with the best cutoff data obtained. Box plots showed that age, sex and alcohol consumption were not correlated with the risk score (**Figure 6A**). However, smoking (**Figure 6B**) did show a correlation with the risk score. Furthermore, risk scores of HPV- HNSCC cases were also evaluated according to their gene signatures as established with the scRNA and TCGA datasets (**Figures 6C, D**). No correlation of the risk scores were found when the cases were divided according to their age and sex as well as alcohol consumption and smoking status. This revealed a specificity of the current gene signature for the assessment of smoking in HPV+ HNSCC patients.

In order to compare the prognosis-correlated factors to those general factors, risk scores for genetic characteristics and clinically-related variables were analyzed by multi-variate Cox regression (**Figures 6E, F**). The forest plots did not show any significance that the current risks examined, thus revealing the significance of the current gene signature and risk scoring system used in this study.

### Profiling the Gene Expression of the HPV+ HNSCC Risk Groups

**Figure 7:**
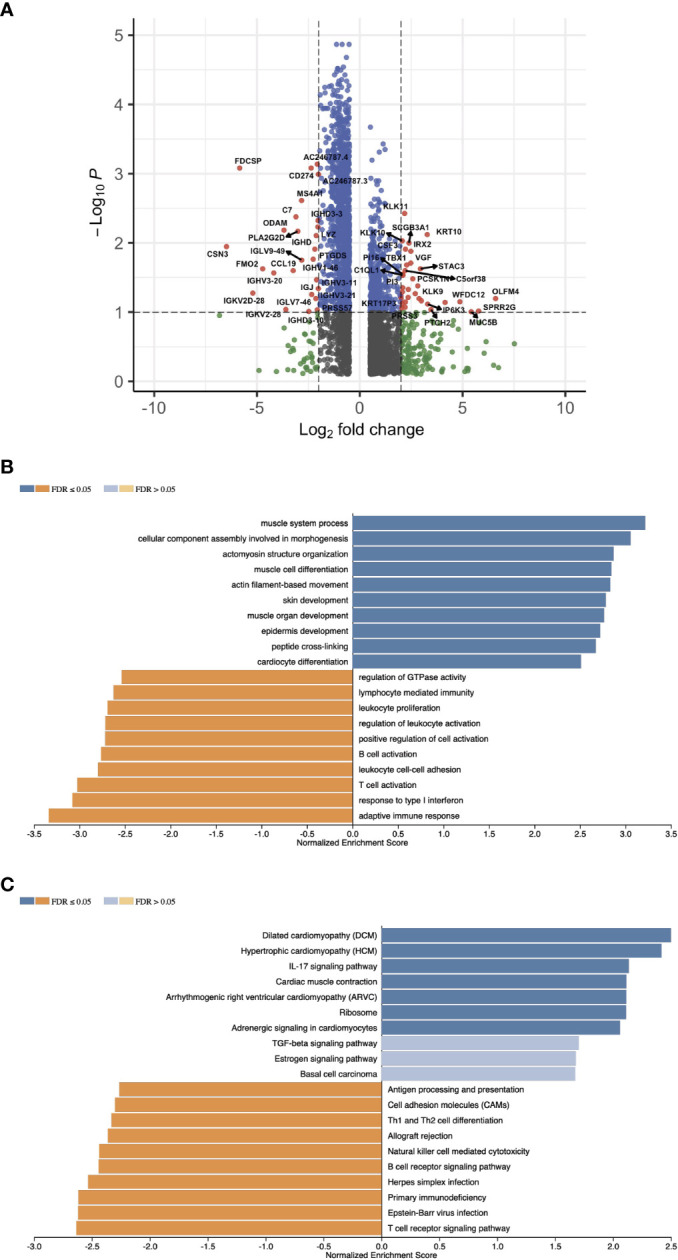
Profiling the gene expression of the HPV+ HNSCC risk groups. **(A)** A volcano plot of the DEGs between high and low risk groups of HPV+ HNSCC TCGA samples. **(B)** Bar graphs showing the enriched gene ontology (GO) Biological Process of the risk DEGs and **(C)** the enriched KEGG pathways.

**Figure 8:**
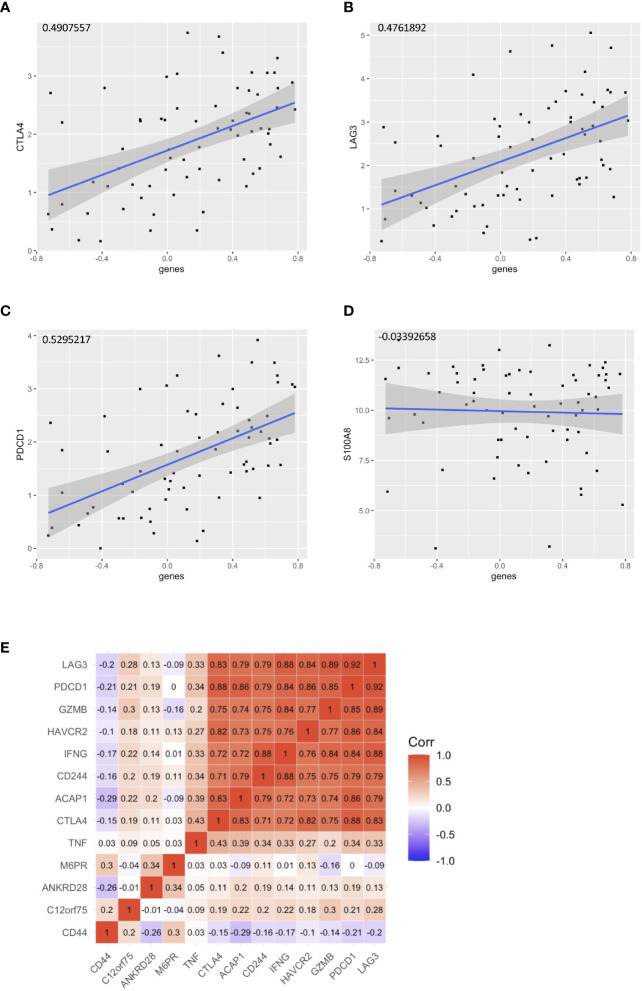
Correlation of the signature genes with the checkpoint related genes. Correlation of the signature genes with **(A)** CTLA4 **(B)**LAG3 and **(C)** PDCD1. **(D)** S100A8 was used as control. **(E)** A corr plot showing the signature genes with the checkpoint related genes.

With the signature genes, we separated the HPV+ HNSCC into high and low risk groups. We investigated the DEGs between high and low risk groups of HPV+ HNSCC TCGA samples (**Figure 7A**). The gene ontology (GO) and KEGG pathways studies showed that the risk DEGs are enriched in the T cell activation and differentiation (**Figures 7B, C**), indicating the involvement of the signature genes in the T cells function. We further investigated the correlation of the signature genes with the immune checkpoint genes and found a decent correlation with CTLA4, LAG3 and PDCD1, but not with the DAMP signal gene S100A8 (**Figures 8A–D**). Especially, a strong correlation was detected between ACAP1 and the checkpoint related genes (**Figure 8E**).

## Discussion

Using a single-variate Cox proportional risk model, we identified that the tumor-infiltration of CD8+ T cells were protective for cases with HPV+ HNSCC, but not for HPV- HNSCC cases. Further, the tumor immunization environment was explored using single-cell sequencing and screened for CD8+ T cell-specific gene feature differences between HPV+ and HPV- HNSCC patients. In addition, we built a prognosis-correlated genetic signature that divided the overall survival of HPV+ HNSCC into two risk groups with the high-risk cases showing poorer prognoses. A prognosis-correlated gene signature consisting of 4 genes with low risk. Then the association between CD8+ T cell genetic traits and clinically related parameters were determined using TCGA dataset and verified in TCGA provisional dataset, to demonstrate the accuracy of genetic traits for prognosis-correlated prediction.

According to the single-cell data, there were significant differences between the immune profiles of HPV- *versus* HPV+ HNSCC patients, which is of significance when designing their immunotherapy regimens. Although the proportion infiltrating CD8 cells were similar in the two HNSCC types, the CD8 gene cell expression profiles were not identical, so more tailored therapies will be required in order to improve the survival rates of patients. The distinct immune profiles in the microenvironment of HPV+ and HPV- HNSCC patients may result from the presence of viral antigens throughout the carcinogenesis process, resulting in the early innate immune responses and the enhancement of the T cells adaptive immune response (20, 21). Further comparisons of the gene transcriptomes of cells with and without the presence of viral antigens throughout the carcinogenesis process is needed to enable the true cause of immune profile differences seen in the presence and absence of HPV in HNSCC patients.

In line with our current results, a recent study noted that between nearly 10% of the infiltrating T cells present in HNSCC were such CD8-positive T cells that target HPV and express PD-1 (22). One of these cells has stem cell characteristics and can expand in large numbers to treat head and neck cancer if cancer immunotherapy is used. Current conventional treatments for head and neck cancer include radiotherapy and chemotherapy, but they may affect the number of immune cells in the body. Therefore, better results may be achieved if immunotherapy is used first and then combined with conventional modalities.

The shortcoming of our current study is the small size of the test group, which, although showing a good trend, somehow lacks statistical significance. Further studies are needed to examine not only HPV+ samples but also specific epitopes of HPV infection.

## Data Availability Statement

The original contributions presented in the study are included in the article/**Supplementary Material**. Further inquiries can be directed to the corresponding authors.

## Ethics Statement

The studies involving human participants were reviewed and approved by Youjiang Medical University for Nationalities. The patients/participants provided their written informed consent to participate in this study.

## Author Contributions

JS and HL designed this study. YW, LM, KC, and JZ analyzed the scRNA-seq data. HL, SH, and JS analyzed the bulk sequencing data. QW, ZW, and SS contributed to scRNA-seq data and helped organize this manuscript. All authors contributed to the article and approved the submitted version.

## Funding

This research work was funded by the National Natural Science Foundation of China (#31970745).

## Conflict of Interest

The authors declare that the research was conducted in the absence of any commercial or financial relationships that could be construed as a potential conflict of interest.

## Publisher’s Note

All claims expressed in this article are solely those of the authors and do not necessarily represent those of their affiliated organizations, or those of the publisher, the editors and the reviewers. Any product that may be evaluated in this article, or claim that may be made by its manufacturer, is not guaranteed or endorsed by the publisher.
